# Brain Oscillations and Predictive Processing

**DOI:** 10.3389/fpsyg.2012.00416

**Published:** 2012-10-17

**Authors:** Juliana Yordanova, Vasil Kolev, Roumen Kirov

**Affiliations:** ^1^Institute of Neurobiology, Bulgarian Academy of SciencesSofia, Bulgaria

The hierarchical generative model presented in the target article is based on theoretical and computational grounds. However, providing neurophysiologic evidence for the presence of distinct neural mechanisms mediating top-down predictions and bottom-up transmission of prediction error is critical for model validity.

In human cognitive electrophysiology, several event-related potentials (ERPs) are related to the mechanisms of comparing incoming with expected events. These are the mismatch negativity (MMN), error-related negativity (ERN), and feedback-related negativity (FRN) operating basically in the sensory, action/performance, and cognitive domains, respectively. These ERP components are all correlated with generating and transmitting mismatch information (Falkenstein et al., 1991; Nieuwenhuis et al., 2004; Näätänen et al., 2007) and have been previously targeted as markers of bottom-up prediction error (Garrido et al., 2009; Cavanagh et al., 2010; Friston, 2012). There are, however, several issues that may question the suitability of these components for testing the predictive coding model.

The latencies of mismatch ERP components are relatively long (150–250 ms for MMN, 200–300 ms for FRN, and around 100 ms for ERN) under the assumption that they reflect a feed-forward “error” input from lower processing levels to the cortex. More critically, there is experimental evidence that much earlier ERP components are sensitive to deviance. For example, brainstem and middle-latency components within 10–80 ms (Slabu et al., 2010, 2012), the P1 component within 10–100 ms (Bendixen et al., 2009), and early gamma (>30 Hz) oscillatory responses at around 50 ms of auditory ERPs (Schadow et al., 2009) discriminate deviant from standard stimuli. Further, the sources of auditory MMN have been localized in the bilateral temporal and in the prefrontal cortices (Giard et al., 1990; Rinne et al., 2000), the latter supposed to mediate a switch/re-allocation of attention focus (Escera et al., 1998, 2003). Likewise, the sources of ERN and FRN are localized in the medial frontal and prefrontal brain regions (Ullsperger and von Cramon, 2001; Holroyd et al., 2004; Cohen et al., 2011). These data show that the “error” negativities of human ERPs may not represent the virtual error information transmitted to the cortex from lower processing levels. Rather, they may reflect a follow-up utilization of the mismatch information that would be incorporated to update and optimize top-down representations (via *attention*, *memory*, and *emotional activations*). Thus, the feed-forward “error” signal presumed by the predictive coding theory should be sought in much earlier time windows after stimulus and even before stimulus (Romei et al., 2010). Referring to the classical “error” or “prediction-violation” ERP components needs to be conceptually clarified.

In search for error prediction ERP signatures, it is important to recognize that ERP components have a complex heterogeneous structure composed of frequency-specific oscillations (Kolev et al., 1997; Yordanova et al., 2000). The ERN, in particular, comprises at least two oscillatory sub-components that carry error signals from co-existent processing systems (theta – 4–7 Hz and delta – <4 Hz) operating in parallel at the level of movement and behavioral control (Yordanova et al., 2004). It is remarkable that the oscillatory theta component has been detected not only for ERN (Luu and Tucker, 2001; Yordanova et al., 2004; Cavanagh et al., 2009, 2010), but it has also been established as a primary constituent of MMN (Fuentemilla et al., 2008; Hsiao et al., 2009; Ko et al., 2012) and FRN (Cohen et al., 2007, 2011; Marco-Pallares et al., 2008; Christie and Tata, 2009). Thus, the oscillatory theta activity may be a consistent neurophysiologic marker of the utilization of the mismatch (error) information by the executive-control systems in the cortex.

In contrast, there is evidence that increased fast-frequency (beta/gamma – 15–80 Hz) oscillations may signal match detection. Cohen et al. (2007) and Marco-Pallares et al. (2008) demonstrated that positive feedback (prediction confirmation) was associated with increased beta/gamma activity, in contrast to negative feedback characterized by enhanced theta oscillations. Schadow et al. (2009) showed that early phase-locked gamma-band responses (20–80 Hz) were significantly increased for predictable regular auditory stimuli as compared to deviant stimuli, as found for fast-frequency responses in the brainstem (Slabu et al., 2012). This is in line with a model according to which matching between incoming stimulus and a memory template results in enhanced gamma-band activity (Herrmann et al., 2004). Thus, time-frequency decomposition of brain responses may be critical for identifying error signals in functional systems operating in parallel at different processing levels.

In the context of the generative predictive model, a major focus of exploration needs to be placed on error processing in different brain states, in which top-down executive-control mechanisms are reduced, totally inhibited, or qualitatively altered. Such states are best represented by different stages of sleep, anesthesia, coma, and brain diseases. For example, neuroimaging studies have demonstrated that subcortical regions and extrastriate visual cortices are more active during rapid eye movement (REM) sleep compared with wake and non-REM sleep, whereas executive-control (dorsolateral prefrontal and parietal) and primary visual cortices are suppressed in REM sleep compared with wake; In non-REM sleep, the activity of all brain areas relevant for information processing during wake is mostly suppressed (Maquet et al., 1996; Braun et al., 1997, 1998; Nofzinger et al., 1997). Interestingly, although inconsistent and contradictory, there is some evidence that in REM sleep, an analog of the MMN emerges despite the gross suppression of both executive top-down processing and external input transmission (Atienza et al., 2000; Ibáñez et al., 2009). No MMN has been reliably recorded during non-REM sleep and slow wave sleep (Ibáñez et al., 2009; Sculthorpe et al., 2009). It remains poorly understood if and how brain states differing dramatically in top-down and bottom-up mechanisms, neurochemistry, synaptic connectivity, and neuroelectric signaling such as sleep stages (Hobson et al., 2000; Hobson and Pace-Schott, 2002; Nir and Tononi, 2010) affect deviance detection, if inherent (free of executive-control prediction) error signals can be generated, and the extent to which the proposed dynamic Bayesian predictions are moderated by consciousness. Probing generative prediction theory would certainly benefit from referring to such models.
